# Chronic Hyperglycemia Compromises Mitochondrial Function in Corneal Epithelial Cells: Implications for the Diabetic Cornea

**DOI:** 10.3390/cells11162567

**Published:** 2022-08-18

**Authors:** Natalia Mussi, Whitney L. Stuard, Jose Marcos Sanches, Danielle M. Robertson

**Affiliations:** Department of Ophthalmology, UT Southwestern Medical Center, Dallas, TX 75390, USA

**Keywords:** hyperglycemia, metabolism, cornea, diabetes, mitochondria

## Abstract

Mitochondrial dysfunction is a major pathophysiological event leading to the onset of diabetic complications. This study investigated the temporal effects of hyperglycemia on mitochondrial metabolism in corneal epithelial cells. To accomplish this, human telomerase-immortalized corneal epithelial cells were cultured in a defined growth medium containing 6 mM glucose. To simulate hyperglycemia, cells were cultured in a medium containing 25 mM D-glucose, and control cells were cultured in mannitol. Using metabolic flux analysis, there was a hyperosmolar-mediated increase in mitochondrial respiration after 24 h. By day 5, there was a decrease in spare respiratory capacity in cells subject to high glucose that remained suppressed throughout the 14-day period. Although respiration remained high through day 9, glycolysis was decreased. Mitochondrial respiration was decreased by day 14. This was accompanied by the restoration of glycolysis to normoglycemic levels. These changes paralleled a decrease in mitochondrial polarization and cell cycle arrest. Together, these data show that chronic but not acute hyperglycemic stress leads to mitochondrial dysfunction. Moreover, the hyperglycemia-induced loss of spare respiratory capacity reduces the ability of corneal epithelial cells to respond to subsequent stress. Compromised mitochondrial function represents a previously unexplored mechanism that likely contributes to corneal complications in diabetes.

## 1. Introduction

The corneal epithelium is a five- to seven-cell-layered stratified epithelial sheet that is contiguous with the limbal and conjunctival epithelia. Together, these three cell types form the ocular surface. Diabetes-related alterations in the corneal epithelium have been well described and occur in up to 70% of patients with diabetes [1,2,3,4]. These include defects in epithelial barrier function, the reduced adherence of basal cells to the basement membrane, the loss of corneal nerves, and a corresponding reduction in aqueous tear secretion leading to the onset of dry eye disease [3,4,5,6,7,8,9,10,11,12,13]. Impaired wound healing has also been reported [14,15,16,17,18,19,20]. Resurfacing the cornea following trauma or ocular surgery can be clinically challenging as many of these corneas do not respond well to supportive therapy due to corneal nerve loss [3,4,21,22]. Chronic hyperglycemia is known to contribute to these pathophysiological alterations [23]. Indeed, in patients with type 1 diabetes, tight glycemic control remains critical for the prevention of diabetic complications [24]. In type 2 disease, however, early glycemic damage due to delays in diagnosis coupled with insulin resistance and dysregulated signaling pathways leads to a high complication rate despite the subsequent attainment of adequate glycemic control [25,26].

Although glucose is present in the tear fluid that bathes ocular surface epithelia, the corneal epithelium is supplied by glucose from the aqueous humor within the anterior segment of the eye. Recent work has shown that the glucose levels in the aqueous correlate with blood glucose levels [27,28]. Poor glycemic control has also been shown to further increase glucose levels in the aqueous. As a hydrophilic molecule, glucose requires a membrane carrier protein to cross the plasma membrane. In the corneal epithelium, this is accomplished through the glucose uptake transporter 1 (GLUT1) [29]. GLUT1 is known for regulating basal glucose uptake in certain tissues in an insulin-independent manner [30]. Early studies evaluating GLUT1 activity in corneal epithelial cells reported that during wound healing, the number of GLUT1 transporters is upregulated to facilitate an increase in glucose uptake [29]. A further comparison between diabetic and non-diabetic corneas revealed little to no difference in GLUT1 expression [31]. Together these findings eliminated GLUT1 as a negative mediator of wound healing in the diabetic cornea.

Diabetes is a complex and multifactorial disease and is associated with changes in cellular metabolism, where mitochondrial dysfunction is a central feature underlying the pathophysiology of many diabetic complications [32,33,34,35]. Mitochondria are endosymbiotic organelles responsible for energy production in the form of ATP, apoptosis, the regulation of cytosolic calcium, and the generation of reactive oxygen species [36,37]. Although the impact of diabetes on mitochondrial and metabolic function has been described in several tissues, including the corneal endothelium, the effects of hyperglycemic stress on mitochondrial and metabolic function in the human corneal epithelium are unknown [38,39,40]. Based on the hypothesis that hyperglycemia compromises mitochondrial respiration in corneal epithelial cells, in the current study, we performed a systematic examination of the effects of acute and chronic hyperglycemia on corneal epithelial cell metabolism.

## 2. Materials and Methods

### 2.1. Cell Line

A human telomerized corneal epithelial (hTCEpi) cell line previously developed and characterized by our laboratory, was used for these studies [41]. hTCEpi cells were cultured in a serum-free keratinocyte growth medium (KGM) containing 0.06 mM calcium supplemented to 0.15 mM (VWR, Radnor, PA, USA), 6 mM glucose, bovine pituitary extract (0.0004 mL/mL), recombinant human epidermal growth factor (0.125 ng/mL), recombinant human insulin (5 μg/mL), hydrocortisone (0.33 μg/mL), epinephrine (0.39 μg/mL), and human holo-transferrin (10 μg/mL, PromoCell; VWR, Radnor, PA, USA). For all experiments, cells were cultured at 37 °C in 5% CO_2_. Hyperglycemic stress was induced by the addition of 19 mM of D-glucose (Sigma-Aldrich, St. Louis, MO, USA), and 19 mM of mannitol was used as an osmotic control (Sigma Aldrich). Test and control cells were cultured for 24 h, 5, 7, 9, or 14 days.

### 2.2. Mitochondrial Measurements

Mitochondrial membrane polarization was measured using tetraethyl-benzimidazolyl-carbocyanine iodide (JC-1) dye (Invitrogen/Molecular Probes, Eugene, OR, USA). hTCEpi cells were cultured in growth media supplemented with or without high glucose or mannitol for 14 days. Cells were then seeded onto 35 mm glass-bottom dishes (MatTek Corporation, Ashland, MA, USA) and allowed to adhere overnight. Cells were then incubated at 37 °C with 10 μg/mL of JC-1. After incubation, cells were washed three times in PBS and imaged on a Leica SP8 laser scanning confocal microscope (Leica Microsystems, Heidelberg, Germany) using a 63× oil objective. The microscope was enclosed within an environmental chamber that maintained cells at 5% CO_2_ and 37 °C during imaging. J-aggregates (multimers) were scanned using a 488 nm excitation laser. J-monomers, indicating mitochondrial polarization, were scanned using a 568 nm laser. All images were sequentially scanned to avoid spectral crosstalk between channels. The entire experiment was repeated two additional times.

### 2.3. Real-Time Metabolic Studies

Real-time measurements of cellular oxygen consumption rate (OCR) and extracellular acidification rate (ECAR) were performed using a Seahorse Metabolic Analyzer XFp (Agilent Technologies, Santa Clara, CA, USA). hTCEpi cells were cultured in a high-glucose medium for 24 h, 5, 7, 9, or 14 days and then seeded onto Seahorse XFp mini plates at a concentration of 30,000 cells per well and cultured overnight. Prior to initiating measurements, cells were first incubated for 1 h at 37 °C in a Seahorse XF Dulbecco’s modified Eagle’s medium (DMEM) containing 5 mM 4-(2-hydroxyethyl)-1-pipera-zineethanesulfonic acid (HEPES, pH 7.4) and supplemented with 1 mM pyruvate, 2 mM glutamine, and 10 mM glucose in a non-CO_2_ incubator. OCR and ECAR were analyzed using a Seahorse XFp Cell Mito Stress Test kit (Agilent Technologies, Santa Clara, CA, USA). All assays were performed according to manufacturer instructions. For the XFp Cell Mito Stress assay, the first injection was 10 μM oligomycin followed by one injection of 5 μM carbonyl cyanide 4-(trifluoromethoxy)phenylhdrazone (FCCP) and a final injection of 5 μM of rotenone/antimycin A (R/A). FCCP and R/A are inhibitors of complex I and III, respectively. This allows for complete inhibition of mitochondrial respiration and determination of non-mitochondrial respiration. Mean OCR and ECAR values were calculated from the measurement obtained at the third time point prior to the addition of any test compounds. The ratio for OCR/ECAR was also calculated for each experiment. Basal respiration was calculated as OCR minus non-mitochondrial oxygen consumption. The proton leak and ATP-linked respiration were also evaluated. Spare respiratory capacity was calculated by subtracting basal respiration from FCCP-stimulated OCR. Data were analyzed using the manufacturer-provided Wave software version 2.3.0. All samples were plated in 6 replicate wells. All outcome measures were normalized to total cell number using a Celigo imaging cytometer (Nexcelom Bioscience, Lawrence, MA, USA). Each experiment was repeated a minimum of two additional times.

### 2.4. Cell Cycle Determination

To assess the acute effects of hyperglycemia on the cell cycle, 1.2 × 10^5^ cells per well were seeded onto 24-well tissue culture plates and grown for 24 h. To evaluate the effects of chronic hyperglycemia on the cell cycle, cells were cultured in T25 tissue culture flasks for 6 or 13 days. Cells were then seeded onto 24-well tissue culture plates and grown for 24 h. After 24 h, cells were fixed using ice-cold ethanol for 15 min at 4 °C and then stained with 300 μg/mL propidium iodide containing RNase (Cell Signaling, Danvers, MA, USA). After washing with phosphate-buffered saline, cells were imaged using a Celigo imaging cytometer (Nexcelom Bioscience, Lawrence, MA, USA). Cell cycle analysis was performed using the manufacturer-provided software. Each experiment was performed in triplicate and repeated a minimum of two additional times.

### 2.5. Cell Proliferation Assay

To assess cell proliferation, hTCEpi cells were seeded at 5 × 10^4^ cells per well in a 6-well culture plate in either keratinocyte growth media, media containing 25 mM D-glucose, or mannitol. Cell numbers were measured daily using a Celigo imaging cytometer (Nexcelom Biosciences, Lawrence, MA, USA) for 7 days. To determine the effects of glucose after 14 days, cells were continuously cultured in 19 mM D-glucose or mannitol in T-25 flasks for 7 days. Cells were then seeded into 6-well culture plates and cultured for an additional 7 days in their respective condition. All samples were plated in triplicate wells and the number of cells was normalized to the KGM control at each time point. Each experiment was repeated a minimum of two additional times.

### 2.6. Cell Migration

Cell migration was measured using a scratch wound assay. To accomplish this, 4 × 10^5^ cells per well were plated in triplicate in a 6-well culture plate after 24 h, 7 days, or 14 days in hyperglycemic culture and allowed to adhere overnight. The scratch was performed using a 200 μL pipette tip, generating a cell-free region. The cell-free region consisted of two perpendicular straight lines, creating a cross in each well. After the scratch, cultures were gently washed to remove detached cells using KGM media [42]. The cells were imaged using the Celigo at time zero, 6 h, and 24 h. The images were processed using MetaMorph Software (Molecular Devices, Sunnyvale, CA, USA). The distance from the cross to the point of measurement was held constant across all wells. Percent closure was quantified by determining the area between the cell borders within a defined region. Measurements were normalized to the area between cell borders at time zero immediately following the scratch for each well. Each experiment was repeated a minimum of two additional times.

### 2.7. Statistical Analysis

All data are expressed as mean ± standard deviation. Normality was assessed using a Shapiro–Wilks test. For comparison of an outcome measure between multiple groups with a normal distribution, a one-way ANOVA was used with an appropriate post hoc comparison test (Student–Newman Kheuls test). For data with a non-normal distribution, a one-way ANOVA on ranks with an appropriate post hoc comparison test was used. Statistical significance was set at *p* < 0.05.

## 3. Results

**Figure 1:**
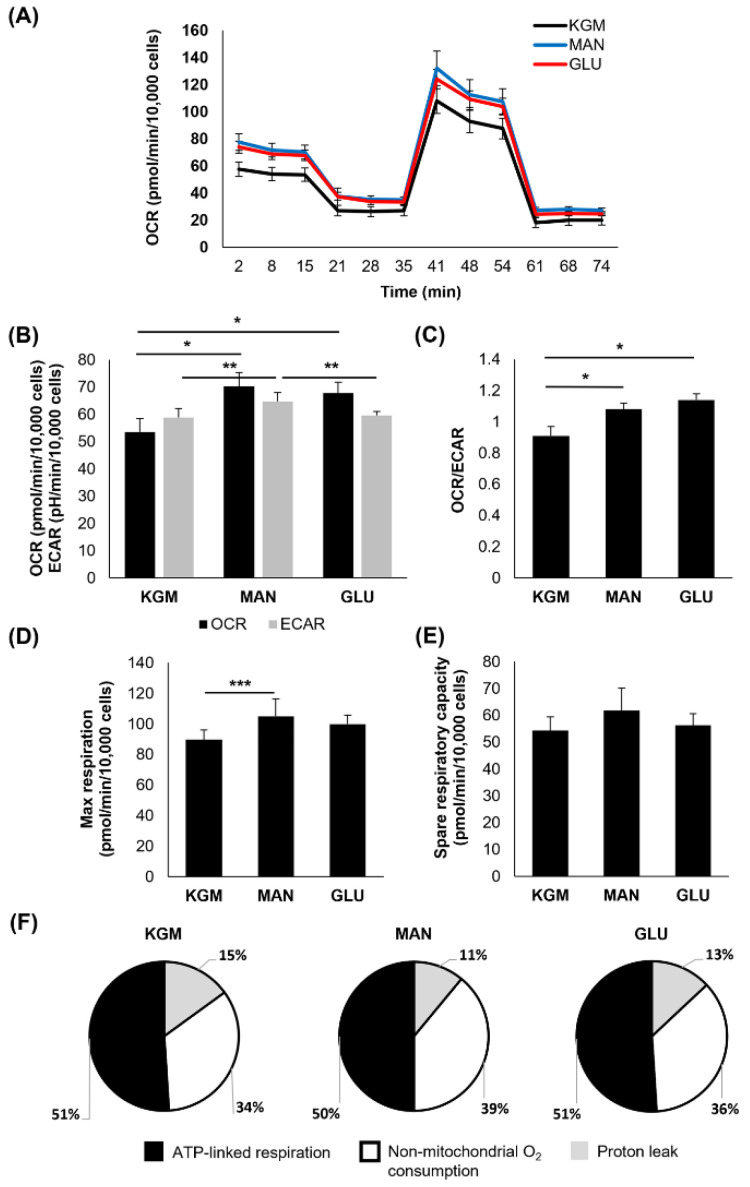
Acute osmotic stress, not hyperglycemia, increases oxygen consumption in corneal epithelial cells. (**A**) OCR plotted as a function of time for both test and control groups. (**B**) OCR was increased in cells treated with 25 mM glucose and 25 mM mannitol compared to the KGM control (* *p* < 0.001). ECAR was increased in cells cultured in mannitol (** *p* = 0.007). (**C**) The OCR/ECAR ratio was increased in glucose- and mannitol-treated cells (* *p* < 0.001). (**D**) Maximal respiration was increased in mannitol-treated cells (*** *p* = 0.016). (**E**) There were no differences in spare respiratory capacity in glucose- or mannitol-treated cells (*p* = 0.129). (**F**) The proportion of oxygen consumption due to ATP-linked respiration, non-mitochondrial oxygen consumption, and the proton leak in the KGM control compared to glucose- and mannitol-treated cells. Bar graphs are presented as mean ± standard deviation. Pie charts are presented as percentage of total oxygen consumption. Graphs are representative of one experiment that was repeated three independent times. One-way ANOVA on ranks with Tukey post hoc comparison test (non-mitochondrial oxygen consumption) and one-way ANOVA with SNK multiple comparison test for all others.

To investigate metabolic changes in response to the hyperglycemic culture at the indicated time points, a Mito Stress test was used. At 24 h, the earliest time point, few changes were noted (Figure 1). There was a small but significant increase in the OCR in cells cultured in either D-glucose or mannitol, suggesting that this effect was due to osmotic stress (*p* < 0.001, Figure 1A,B). In contrast to this, the ECAR was slightly increased in the mannitol-treated cells (*p* = 0.007). The increase in the OCR in the cells cultured in high sugar shifted the OCR/ECAR ratio to a more respiratory phenotype (*p* < 0.001, Figure 1C). The cells cultured in mannitol showed an increase in maximal respiration (*p* = 0.016, Figure 1D) that was not evident in the cells cultured with high glucose. There were no measurable differences in spare respiratory capacity between the groups (Figure 1E). Since the OCR is a measure of total cellular respiration comprising ATP-linked respiration, non-mitochondrial respiration, and the proton leak, we next evaluated these parameters both in terms of the proportion that each contributes to the total OCR (Figure 1F) and their actual values (Table 1). Although the overall proportion of ATP-linked respiration that contributed to the total OCR was similar among the test and control groups, ATP-linked respiration was increased in the high-glucose- and mannitol-treated cells compared to the KGM (Table 1, *p* < 0.001), indicating that cells were requiring additional energy in response to hyperosmolar stress. Non-mitochondrial oxygen consumption was also increased in the mannitol-treated cells compared to the KGM (*p* = 0.003). This accounted in part for the increase in the OCR in the mannitol group. There was no difference in the proton leak between the test cells and controls.

**Figure 2:**
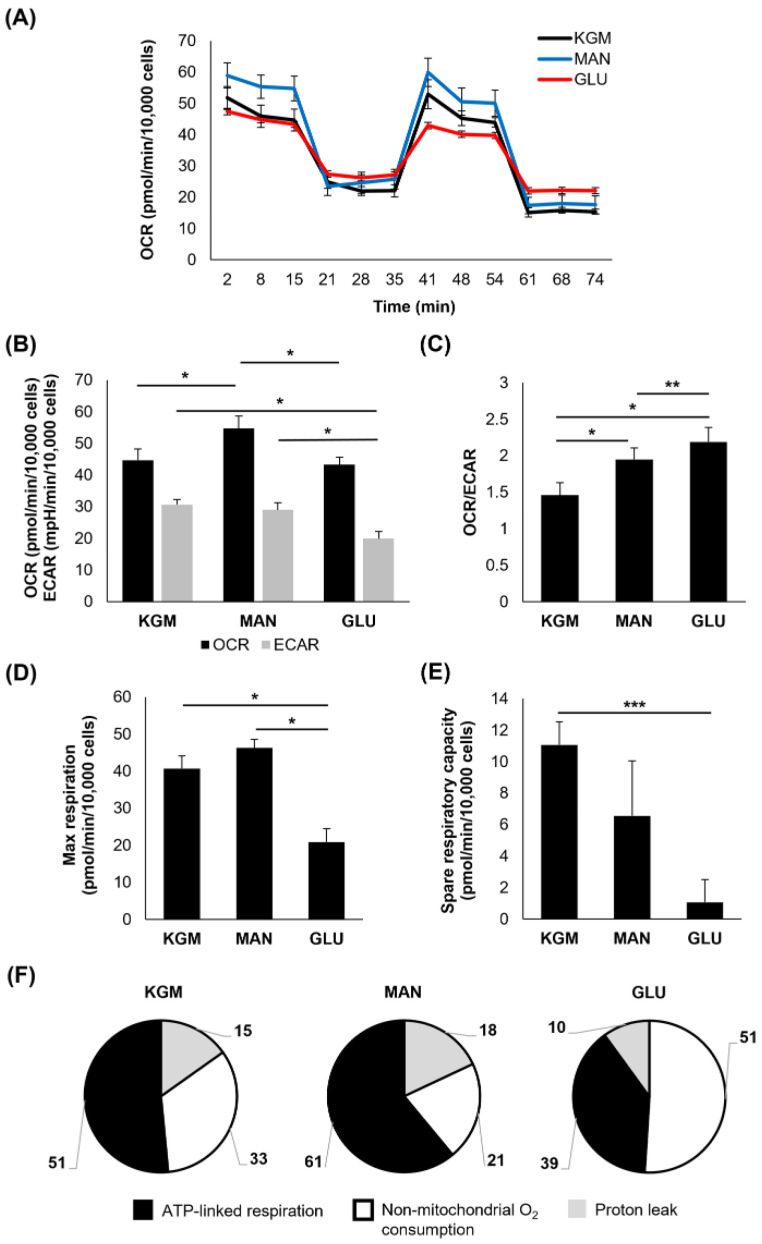
Hyperglycemia decreased both glycolysis and spare respiratory capacity after 5 days of culture. (**A**) OCR plotted as a function of time for both test and control groups. (**B**) OCR was only increased in mannitol-treated cells (* *p* < 0.001). In contrast, ECAR was decreased in cells cultured in high glucose (* *p* < 0.001). (**C**) The OCR/ECAR ratio shifted toward a more respiratory phenotype in cells cultured in high glucose (* *p* < 0.001). Mannitol-treated cells were more respiratory than cells in KGM but did not reach the same threshold as cells cultured in high glucose (** *p* = 0.038). (**D**) There was a decrease in maximal respiration in cells cultured in high glucose (* *p* < 0.001). (**E**) Spare respiratory capacity was similarly decreased in cells cultured in high glucose (*** *p* < 0.05). (**F**) The proportion of oxygen consumption due to ATP-linked respiration, non-mitochondrial oxygen consumption, and the proton leak in the KGM control compared to glucose- and mannitol-treated cells. Bar graphs are presented as mean ± standard deviation. Pie charts are presented as percentage of total oxygen consumption. Graphs are representative of one experiment that was repeated three independent times. One-way ANOVA on ranks with Dunn’s post hoc comparison test (spare respiratory capacity and proton leak) and one-way ANOVA with SNK multiple comparison test for all others.

In contrast to the results at 24 h, metabolic changes were evident after 5 days of culture (Figure 2). Although mannitol maintained an osmotic effect on the OCR (*p* < 0.001, Figure 2A,B), the OCR for the high-glucose-treated cells was unchanged compared to the cells cultured in the KGM (Figure 2B). The ECAR, a surrogate marker for glycolysis, was decreased in the high-glucose-treated cells (*p* < 0.001, Figure 2B). This led to a shift in the OCR/ECAR ratio toward a more respiratory phenotype in both the glucose- and mannitol-treated cells (*p* < 0.001, Figure 2C). In addition to the ECAR, there was a significant decrease in the maximal respiration and spare respiratory capacity in the high-glucose-treated cells (*p* < 0.001 and *p* < 0.05 for maximal respiration and spare respiratory, respectively, Figure 2D,E). As shown in Figure 2F, the cells cultured in high glucose appeared to have a higher percentage of non-mitochondrial oxygen consumption, suggestive of inefficient oxidative phosphorylation. Indeed, there was a decrease in the ATP-linked respiration in the high-glucose-treated cells compared to the KGM and mannitol controls (Table 1, *p* < 0.001). The proton leak was lowest in this group (*p* < 0.001). It should be noted, however, that on repeated runs, the OCR and ATP-linked respiration varied for the high-glucose-treated cells, with some runs still showing increased OCR and ATP-linked respiration similar to the mannitol-treated cells. Thus, the response to high glucose was still due to some level of hyperosmolar stress at this time point. The OCR and ATP-linked respiration were consistently elevated for mannitol-treated cells in all runs (*p* < 0.001).

**Figure 3:**
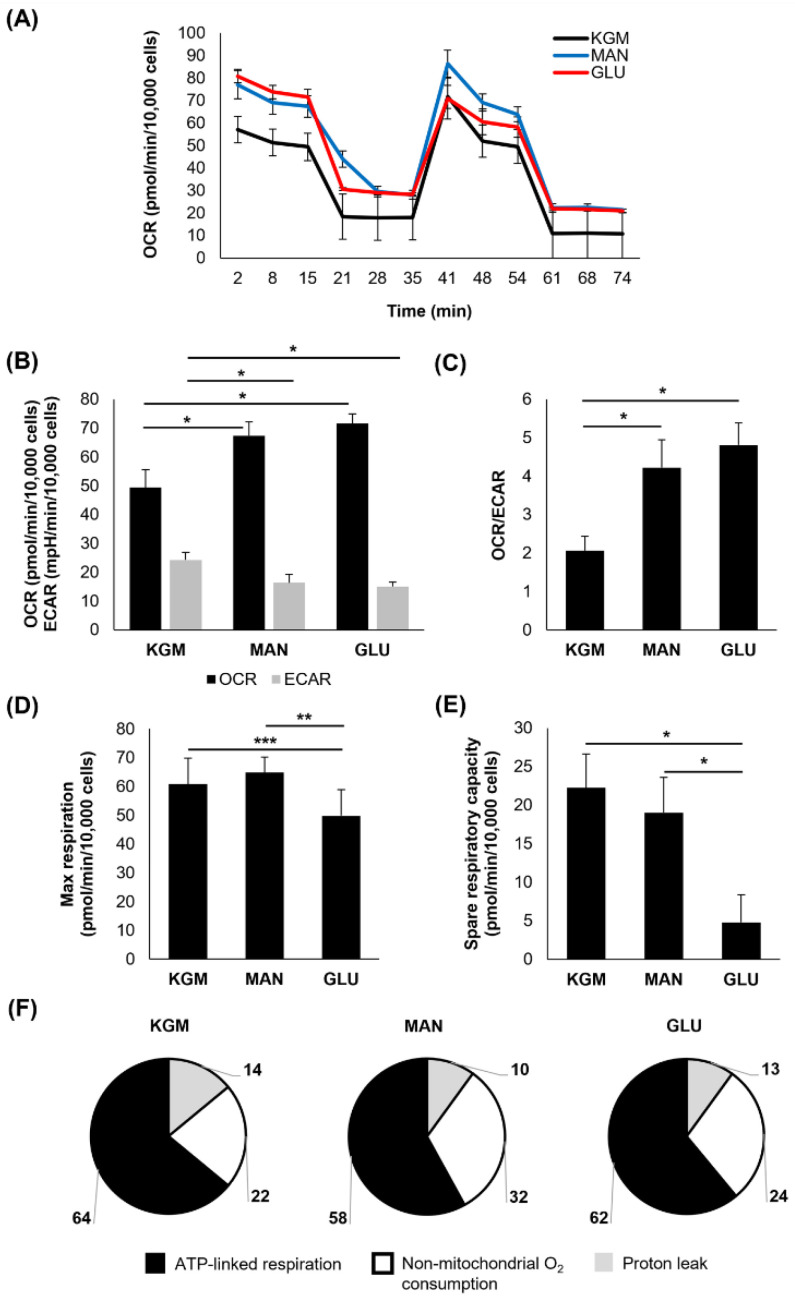
Spare respiratory capacity remained decreased in response to hyperglycemia after 7 days in culture. (**A**) OCR plotted as a function of time for both test and control groups. (**B**) OCR was increased in both glucose- and mannitol-treated cells (* *p* < 0.001), whereas ECAR was decreased compared to KGM (* *p* < 0.001). (**C**) There was a shift toward a more respiratory phenotype for cells cultured in glucose and mannitol (* *p* < 0.001). (**D**) Maximal respiration was decreased in cells treated with high glucose (** *p* = 0.038, *** *p* = 0.014). (**E**) Spare respiratory capacity was decreased only in cells cultured in high glucose (* *p* < 0.001). (**F**) The proportion of oxygen consumption due to ATP-linked respiration, non-mitochondrial oxygen consumption, and the proton leak in the KGM control compared to glucose- and mannitol-treated cells. Bar graphs are presented as mean ± standard deviation. Pie charts are presented as percentage of total oxygen consumption. Graphs are representative of one experiment that was repeated three independent times. One-way ANOVA on ranks with Dunn’s post hoc comparison test (non-mitochondrial oxygen consumption) and one-way ANOVA with SNK multiple comparison test for all others.

After 7 days of culture, the high-glucose-treated cells again showed an increase in the OCR that was accompanied by a drop in the ECAR (*p* < 0.001, Figure 3A,B). This led to an even greater shift in the OCR/ECAR ratio toward a more respiratory phenotype (*p* < 0.001, Figure 3C). The OCR for the mannitol-treated cells remained elevated (*p* < 0.001) and now showed a similar decrease in the ECAR (*p* < 0.001). Similar to the 5-day time point, both the maximal respiration (*p* = 0.014 compared to KGM and *p* = 0.038 compared to mannitol) and spare respiratory capacity were decreased only in the high-glucose cultures (*p* < 0.001, Figure 3D,E). Although the overall proportion of ATP-linked respiration, non-mitochondrial respiration, and the proton leak was similar between the test and control groups (Figure 3F), ATP-linked respiration was high in the cells cultured in high glucose and mannitol (Table 1, *p* < 0.001 high glucose compared to KGM and *p* = 0.004 mannitol compared to KGM). Although non-mitochondrial oxygen consumption trended toward an increase in high glucose, this was only significant in the mannitol-treated cells (*p* < 0.05). There was no difference in the proton leak.

**Figure 4:**
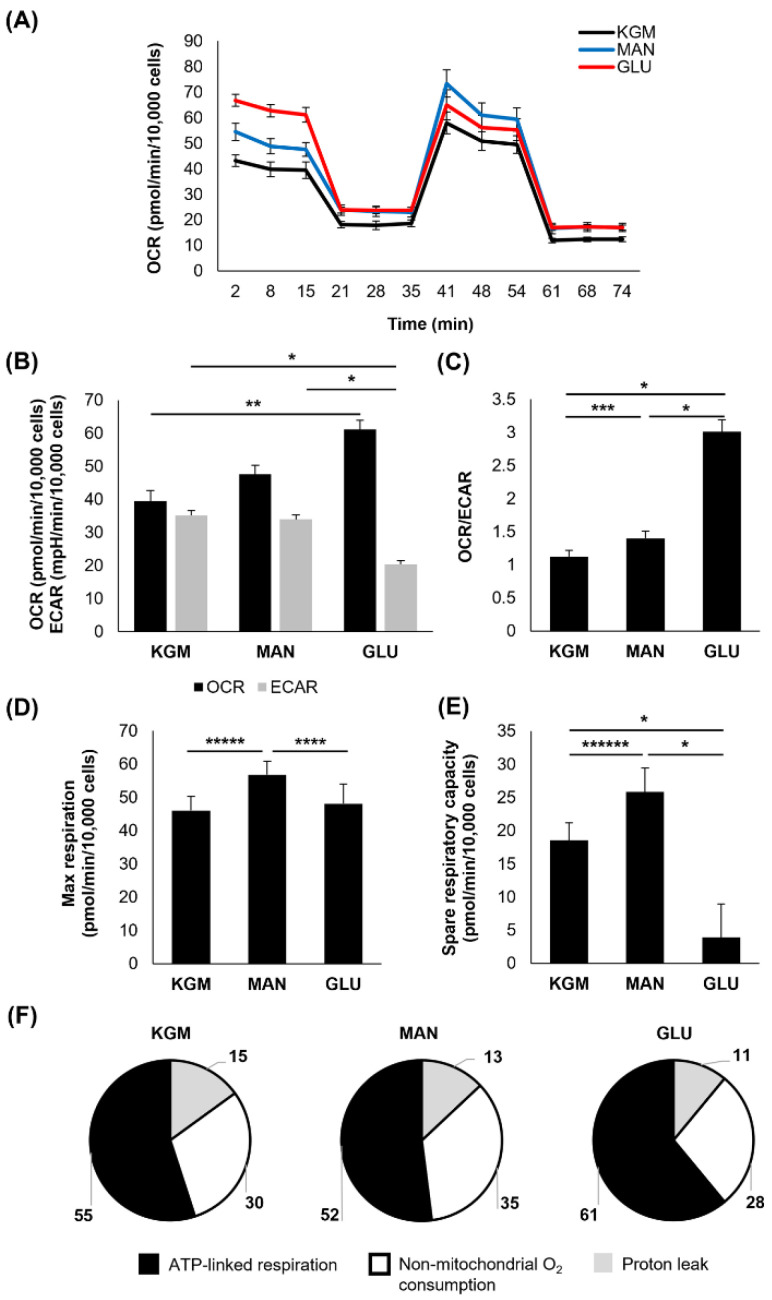
Hyperglycemia, not osmotic stress, shifted cells toward a respiratory phenotype after 9 days of culture. (**A**) OCR plotted as a function of time for both test and control groups. (**B**) OCR was increased in high glucose compared to KGM (** *p* < 0.05), whereas ECAR was decreased in high glucose compared to KGM and mannitol (* *p* < 0.001). (**C**) The OCR/ECAR ratio shifted toward a more respiratory phenotype for cells cultured in high glucose (* *p* < 0.001) and mannitol (*** *p* < 0.003). (**D**) Maximal respiration was increased in mannitol-treated cells compared to KGM (**** *p* = 0.004) and glucose (***** *p* = 0.007). (**E**) Spare respiratory capacity was reduced only in high-glucose-treated cells (* *p* < 0.001, ****** *p* = 0.003). (**F**) The proportion of oxygen consumption due to ATP-linked respiration, non-mitochondrial oxygen consumption, and the proton leak in the KGM control compared to glucose- and mannitol-treated cells. Bar graphs are presented as mean ± standard deviation. Pie charts are presented as percentage of total oxygen consumption. Graphs are representative of one experiment that was repeated three independent times. One-way ANOVA on ranks with Tukey post hoc comparison test (OCR and non-mitochondrial oxygen consumption) and one-way ANOVA with SNK multiple comparison test for all others.

At 9 days of culture, the OCR was increased only in the high-glucose-treated cells (*p* < 0.05 compared to KGM), whereas the ECAR remained decreased (*p* < 0.001, Figure 4A,B). The mannitol-treated cells, however, showed a reduction in the OCR toward the KGM levels. The ECAR similarly increased to the KGM levels in the mannitol-treated cells. This resulted in a more respiratory phenotype for the high-glucose-treated cells (*p* < 0.001), which was no longer evident for the cells in mannitol (Figure 4C). These data suggest that the cells had adapted to the osmotic stress induced by mannitol and that the persistent effects in the high-glucose-treated cells were due to the presence of increased metabolizable D-glucose. The maximal respiration remained elevated only in the mannitol-treated cells (*p* = 0.007 and *p* = 0.004 for mannitol compared to KGM and glucose, Figure 4D). Consistent with days 5 and 7, spare respiratory capacity remained decreased in the high-glucose-treated cells at 9 days (*p* < 0.001, Figure 4E). This continued drop in spare respiratory capacity in the high-glucose-treated cells supports the increase in ATP-linked respiration, as more energy reserves are being used to sustain the cell (*p* < 0.001, Table 1 and Figure 4F). Non-mitochondrial respiration was increased in both the high-glucose- and mannitol-treated cells (*p* < 0.05), indicating inefficient respiration.

**Figure 5:**
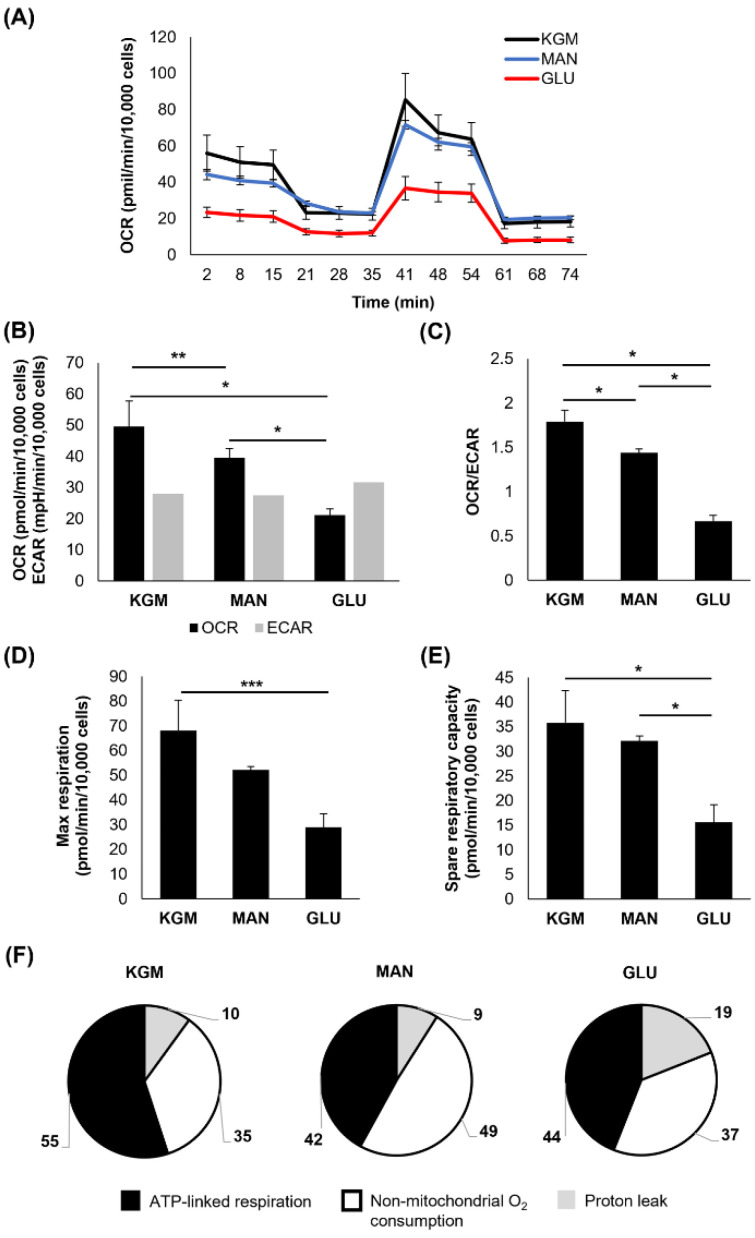
Prolonged hyperglycemia shifted cells toward a more glycolytic phenotype. (**A**) OCR plotted as a function of time for both test and control groups. (**B**) OCR was decreased in high glucose compared to KGM and mannitol (* *p* < 0.001, ** *p* = 0.005). There were no differences in ECAR between groups (*p* = 0.069). (**C**) The OCR/ECAR ratio was shifted toward a more glycolytic phenotype for cells cultured in high glucose (* *p* < 0.001). (**D**) Maximal respiration was decreased in glucose-treated cells (*** *p* < 0.05). (**E**) Spare respiratory capacity was similarly reduced in glucose-treated cells (* *p* < 0.001). (**F**) The proportion of oxygen consumption due to ATP-linked respiration, non-mitochondrial oxygen consumption, and the proton leak in the KGM control compared to glucose- and mannitol-treated cells. Bar graphs are presented as mean ± standard deviation. Pie charts are presented as percentage of total oxygen consumption. Graphs are representative of one experiment that was repeated three independent times. One-way ANOVA on ranks with Dunn’s post hoc comparison test (maximal respiration) and one-way ANOVA with SNK multiple comparison test for all others.

Finally, after 14 days of culture, there were significant reversals in the metabolic phenotype for the cells cultured in high glucose (Figure 5A,B). The OCR levels were decreased for the high-glucose-treated cells compared to the KGM and mannitol (*p* < 0.001, Figure 5B), whereas the ECAR was restored to the KGM levels. Although treatment with mannitol resulted in a partial decrease in the OCR, it remained elevated compared to high glucose (*p* < 0.001). The ECAR was unchanged between the treatment and control groups. These shifts in metabolic activity led to a sharp decline in the OCR/ECAR ratio, supporting a more glycolytic phenotype in the high-glucose-treated cells (*p* < 0.001, Figure 5C). The maximal respiration and spare respiratory capacity remained decreased for the cells cultured in high glucose (*p* < 0.05 and *p* < 0.001 for maximal and spare respiratory capacity, respectively, Figure 5D,E). Differences were present in ATP-linked respiration, non-mitochondrial respiration, and the proton leak (Table 1, Figure 5F). A summary of the metabolic changes is shown in Table 2.

**Figure 6:**
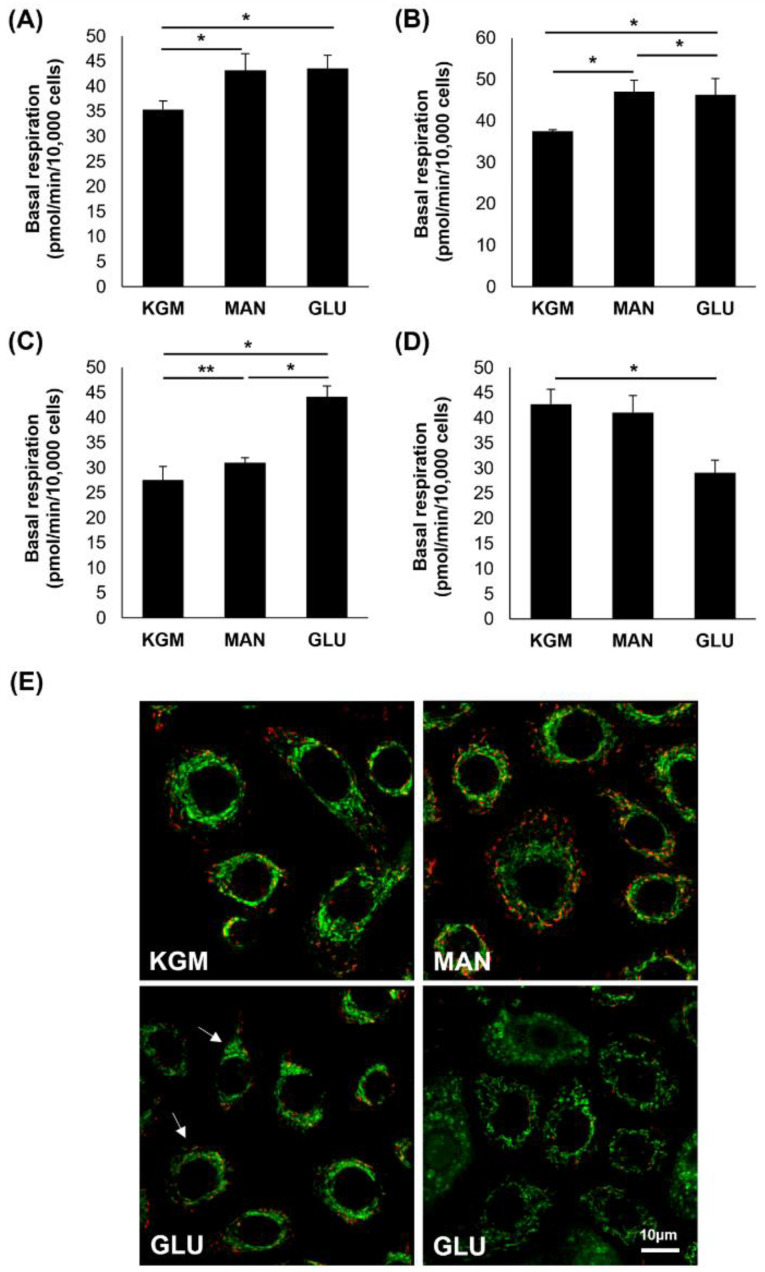
Basal respiration was increased in acute but decreased in chronic hyperglycemic culture. (**A**) Basal respiration showed an early increase in response to osmotic stress at 24 hours (* *p* < 0.001). (**B**) Basal respiration remained increased in response to hyperosmolarity at 5 days (* *p* < 0.001). (**C**) After 9 days, basal respiration was increased in response to hyperglycemia (* *p* < 0.001, ** *p* = 0.011). (**D**) By day 14, basal respiration was significantly decreased in high glucose compared to KGM (* *p* < 0.001). (**E**) After 14 days, JC-1 labeling of cells cultured in KGM showed polarized mitochondria with normal morphology (JC-1 monomers, green; JC-1 aggregates indicating polarization, red). Cells cultured in mannitol were similar to the KGM control. In high glucose, there was a mixed population of cells with decreased polarization and occasional ring-donut-shaped mitochondria (arrows) or more perinuclear accumulation. Scale bar: 10 mm. Images are representative of 3 repeated experiments. Data in graphs are presented as mean ± standard deviation. Graphs are representative of one experiment that was repeated three independent times. One-way ANOVA on ranks with Dunn’s post hoc comparison test (14 days) (**D**) and one-way ANOVA with SNK multiple comparison test for all others.

To further evaluate the effects of high glucose and mannitol on respiration, basal respiration at the third time point prior to the addition of oligomycin was examined. Figure 6A shows an increase in basal respiration in the high-glucose- and mannitol-treated cells after 24 h of culture (*p* < 0.001). At day 5, this increase in basal respiration in the high-glucose- and mannitol-treated cells was still present, confirming that this increase was due to osmotic effects (*p* < 0.001, Figure 6B). By day 9, basal respiration was only increased in the high-glucose-treated cells (*p* < 0.001, Figure 6C), and by day 14, basal respiration was decreased compared to the KGM and mannitol in that same treatment group (*p* < 0.001, Figure 6D). We next investigated the mitochondrial changes at the 14-day time point using the mitochondrial probe JC-1 (Figure 6E). In the KGM control, all cells exhibited polarized mitochondria with normal morphology. In the high-glucose-treated cells, there was a mixed population of cells showing two different mitochondrial phenotypes. Some cells had polarized mitochondria with a smaller, condensed appearance, whereas other cells exhibited mitochondrial depolarization and fragmentation with ring-donut-shaped mitochondria. Mitochondria in the mannitol-treated group remained polarized and had a morphology similar to the KGM control.

**Figure 7:**
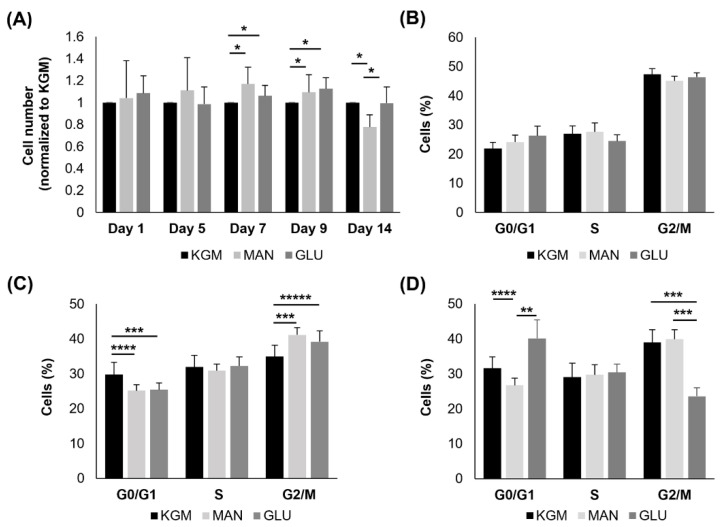
Prolonged hyperglycemia triggered cell cycle arrest after 14 days. (**A**) Cell counts after acute and prolonged culture showed no differences in cell numbers after 24 h or 5 days (*p* = 0.709 and *p* = 0.307 for 24 h and 5 days, respectively). Cell numbers were increased in response to treatment with glucose and mannitol at days 7 and 9. On day 14, cell numbers were decreased in mannitol (* *p* < 0.05). (**B**) Acute exposure (24 h) of culture to high glucose and mannitol had no effect on cell cycle (*p* = 0.568 G0/G1, *p* = 0.159 S, *p* = 0.582 G2/M). (**C**) After 7 days, there was a transient increase in cells cultured in hyperosmolar stress in G2/M (*** *p* = 0.002, **** *p* = 0.004, ***** *p* = 0.008). (**D**) At day 14, there was an increase in cells entering G0/G1 for glucose-treated cells, indicating an arrest at this time point, whereas there were fewer cells entering G0/G1 for mannitol (** *p* < 0.001, *** *p* = 0.005, **** *p* = 0.003). Data are presented as mean ± standard deviation. Graphs are representative of one experiment that was repeated three independent times. One-way ANOVA with SNK multiple comparison test.

To compare metabolic changes with cell function, we next examined the effects of high glucose on cell growth and the cell cycle. As shown in Figure 7A, there were no differences in cell numbers in the high-glucose- and mannitol-treated groups compared to the KGM at 24 h or 5 days. At days 7 and 9, both the high-glucose- and mannitol-treated cells showed a small but significant increase in cell numbers, consistent with the increased OCR (*p* < 0.05). By day 14, there was no longer a decrease in cell numbers in the high-glucose compared to the KGM groups. Cell numbers were decreased in the mannitol-treated cells (*p* < 0.05). In terms of the cell cycle, there were no differences noted after 24 h of culture in high glucose or mannitol (Figure 7B). At 7 days, cells in both the high-glucose and mannitol groups showed evidence of cell cycle arrest or slowing down at G2/M (*p* = 0.008 and *p* < 0.001 for high glucose and mannitol, respectively, compared to KGM, Figure 7C). Contrary to this, by day 14, there were no signs of cell cycle arrest at G2/M (Figure 7D). Instead, cells cultured in high glucose were arrested in G0/G1 (*p* = 0.003 for high glucose compared to KGM and *p* < 0.001 for high glucose compared to mannitol). Fewer cells were entering G0/G1 in the mannitol group.

**Figure 8:**
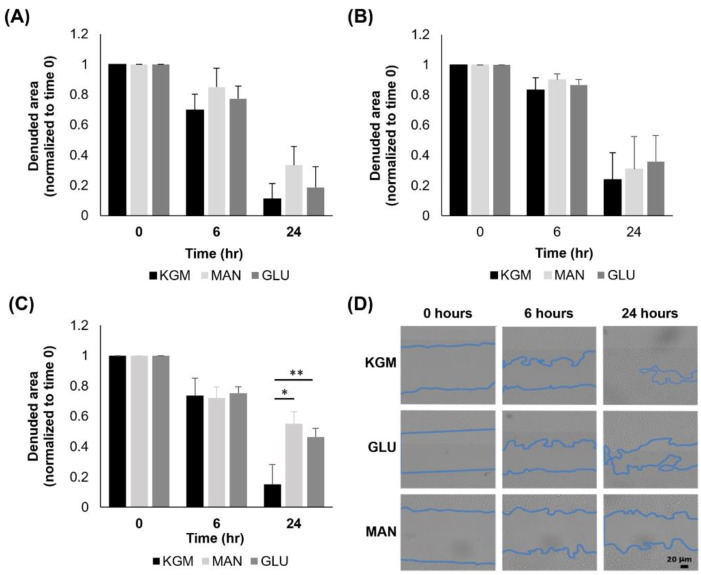
Cell migration was impeded by hyperosmotic stress. (**A**) There was no measurable difference in the rate of cell migration in cells exposed to hyperosmolar or hyperglycemic stress for 24 h (*p* = 0.179 at 6 h, *p* = 0.154 at 24 h). (**B**) There were no differences in migration after 7 days of culture in high glucose or mannitol (*p* = 0.0343 at 6 h, *p* = 0.905 at 24 h). (**C**) Prolonged (14 days) hyperosmolar stress-impaired cell migration at 24 h (*p* = 0.197 at 6 h, * *p* < 0.001 and ** *p* = 0.02 at 24 h). (**D**) Phase contrast imaging showing cell migration over 24 h. Scale bar: 20 mm. Data are presented as mean ± standard deviation. Graphs are representative of one experiment that was repeated three independent times. One-way ANOVA, SNK multiple comparison test.

Lastly, we analyzed cell migration using a scratch assay with cells cultured for either 24 h, 7 days, or 14 days in high glucose or mannitol and then seeded onto 6-well plates. Cells were analyzed immediately after the scratch and again at 6 h and 24 h. There were no changes in migration at either 24 h (Figure 8A) or 7 days (Figure 8B) of high glucose culture. At day 14, cultures in high glucose and mannitol both decreased cell migration compared to the KGM control (Figure 8C, *p* = 0.002 and *p* < 0.001 for KGM compared to glucose and mannitol, respectively). This indicates that the effect on cell migration was due to hyperosmolarity and not hyperglycemia. The representative images of cells cultured for 14 days and subject to the scratch assay are shown in Figure 8D.

## 4. Discussion

In this study, we performed a comprehensive analysis of mitochondrial and glycolytic metabolic changes in corneal epithelial cells exposed to acute and chronic hyperosmolar stress. The first key finding in this study, and of high relevance to clinical disease, is the early decrease in spare respiratory in cells cultured in high levels of glucose. Not only was spare respiratory capacity decreased after only five days of hyperglycemic culture, but it remained decreased throughout the full fourteen days. Only metabolizable glucose and not mannitol led to a reduction in the spare respiratory capacity, confirming that this was not due to hyperosmolar stress. In agreement with this, similar findings were shown in corneal endothelial cells harvested from advanced diabetic human donors [38]. Likewise, outside the eye, work carried out in kidney cells and streptozotocin-treated rats has also found hyperglycemia-induced changes in mitochondrial bioenergetics and a decrease in spare respiratory capacity [40,43]. As the main energy reserve for a cell, spare respiratory capacity is an integral factor in the ability of a cell to respond to stress. Without it, mitochondria are unable to meet the increased energy demand, leading to ATP crisis, cellular compromise, and death. A reduction in spare respiratory capacity has also been associated with increased sensitivity to oxidative stress [44]. Not surprisingly, a decrease or loss of the spare respiratory capacity has been associated with multiple disease pathologies including diabetes [39,40,44,45]. Since spare respiratory capacity is a measure of mitochondrial fitness, the reduction in spare respiratory capacity reported in this study would suggest that chronic, hyperglycemic stress leads to mitochondrial compromise in corneal epithelial cells.

In mammalian cells, mitochondria serve as critical stress response elements that mediate the decision between adaptation and cell death. Despite being a stratified, differentiated epithelium with a relatively low mitochondrial content, corneal epithelial cells are highly sensitive to osmotic changes. Indeed, our prior studies investigating the effects of high levels of salt on corneal epithelial cells found that low levels of salt drive respiration, whereas higher levels of salt decrease it [46]. Consistent with this work, here we show that mild, acute hyperosmotic stress induced by elevated levels of both glucose and mannitol increases mitochondrial respiration. This osmotic stress-induced increase in respiration provides the cell with the necessary resources to respond to a new metabolic demand. As the stress is prolonged, increased respiration is maintained despite a drop in glycolysis. This bioenergetic phenotype is sustained until mitochondria begin to depolarize and fragment. This induces a fuel switch, shifting cells toward a glycolytic phenotype. This shift in glycolysis represents a double-edged sword. Although it may help with glucose consumption and a subsequent reduction in hyperosmolarity in the extracellular environment, the decrease in ATP generation due to the loss of sufficient mitochondrial activity may leave the cell with a substantial energy deficit.

In terms of overall cell growth, the hyperosmotic increase in respiration appeared to coincide with an increase in cell numbers after 7 days in culture despite evidence of a transient cell cycle arrest or slowdown in G2/M. The mechanism by which the cells were able to push through this is unclear but likely suggests it is adaptive in nature. Recent data investigating hyperosmotic shock in yeast have demonstrated that stress-induced changes in the cell cycle function in effect as a carbon valve to allocate the appropriate nutrient supply [47]. This biphasic response was also evident in our hyperosmolarity studies investigating the effects of salt on corneal epithelial cells [48]. Similar to our salt studies, both glucose and mannitol induced this effect, confirming a role in hyperosmolarity. In contrast to this, the subsequent arrest in G0/G1 after 14 days of high glucose is consistent with the loss of mitochondrial polarization that was seen in our JC-1 staining and the drop in the basal OCR. Thus, cells may not be able to generate the energy that is needed for entry into the S phase. Since cell cycle arrest is directly related to proliferation, these data indicate that cells exposed to chronic hyperglycemia do not have sufficient ATP to drive mitosis, which is needed to sustain homeostasis.

One of the surprising findings in this work was the lack of robust changes in corneal epithelial cell growth and migration in response to hyperglycemia. Over the 14-day period tested, we were unable to show a hyperglycemic-specific effect using a scratch assay. Instead, we found that hyperosmolar stress negatively impacted migration. The impact of hyperglycemia on cellular migration in the published literature is equivocal. Although a recent report evaluating the effects of high glucose on corneal and conjunctival epithelial cells reported similar findings to ours, others have found a hyperglycemia-induced delay in migration and growth [49,50]. Culture conditions and the cell type used may impact the cellular response to hyperglycemia and hyperosmolarity. In the present study, our telomerase-immortalized human corneal epithelial cell line was used. The potential for cell lines to maintain a relatively low spare respiratory capacity has been addressed in a prior review [51]. This phenotype is also seen in certain types of primary cultures. This is due to the proliferative demand in cell culture and the need to tap into energy reserves. Since corneal epithelial cells are known to be highly glycolytic, the need to draw on their mitochondrial reserve in culture would be expected. In contrast to this, differentiated cells often possess a much higher spare respiratory capacity due to the lack of ATP that is needed for growth. Moreover, many factors influence spare respiratory capacity including mitochondrial dynamics and quality control such as mitophagy. In our prior studies, we have shown that the hTCEpi cell line has a stable genome and maintains high levels of ΔNp63 when in a proliferative state [41,52]. We have further shown that these cells retain normal differentiation characteristics [41]. The presence of insulin in culture media is also a consideration since insulin is mito-protective and mediates mitochondrial activity and mitophagy through mTOR signaling [53]. Finally, we have found that mitochondrial activity, mitophagy, and dynamics are highly repeatable in both our cell line and primary cultured corneal epithelial cells [46,53,54]. Further investigation is now needed to fully translate these findings to the in vivo ocular surface.

One final interesting yet unexplainable finding in this study is the change in mitochondria polarization in the mannitol-treated cells. The shift in mitochondrial morphology and depolarization in 14-day glucose-treated cells is consistent with the decrease in the OCR that was reported at this time point. Depolarization would promote PINK1 accumulation at the outer mitochondrial membrane and mitophagy would ensue. This would allow for the clearance of the damaged portions of the organelle and explain why the cells are not undergoing frank cell death. In contrast to glucose, mannitol increased the number of JC-1 monomers, suggesting increased polarization. This did not appear to correlate with a change in spare respiratory capacity or basal respiration. In certain cell types, mitochondria have been shown to spontaneously polarize following sublethal stress [55]. In the hTCEpi cell line and primary corneal epithelial cells, we have shown that mild stress induced by growth factor withdrawal drives mitochondrial depolarization and that concurrent treatment with insulin not only blocks depolarization but increases the overall polarization state [53]. This is associated with the elongation of the mitochondrial network, similar to what is seen here in the mannitol-treated cells. We have further found that siRNA knockdown of the insulin receptor in these same cells drives robust ring-donut fragmentation with occasional cells demonstrating massive hyperpolarization. In this latter case, we believe that this massive hyperpolarization occurs prior to the onset of mitochondrial fragmentation. Although further studies are needed to understand the mechanism behind the mannitol-mediated increase in polarization, we hypothesize that this change is due to mild hyperosmolar stress and is not a mechanism for the induction of cell death. This, however, remains to be proven.

A few limitations exist with respect to the current study design. These include the use of the ECAR as a surrogate marker for glycolysis and the absence of corresponding in vivo measurements. The latter is particularly important and warrants further study as factors other than frank hyperglycemia are involved in the onset of diabetic keratopathy, particularly in patients with longstanding or uncontrolled disease. This includes a loss of corneal nerves, changes in the basement membrane, the accumulation of advanced glycation end products, and epigenetic modifications [1,3,5,14,56]. Moreover, more than half of patients with diabetes present with some level of dry eye, increasing the likelihood of a hyperosmolar tear film, which can further damage the corneal surface [6]. Thus, a decrease in proliferation is only one aspect of the cellular response that has gone awry in diabetes. The absence of inflammation in vitro may also impact these findings. Despite these limitations, these data support that chronic and not acute exposure to high glucose is required to induce mitochondrial compromise in corneal epithelial cells. The inefficient production of ATP by glycolysis coupled with the decrease in spare respiratory capacity leaves the cornea susceptible to acute stress in the presence of increased energy demand. As a stratified tissue that faces the external environment and is subject to chronic stress, a sufficient ATP supply is essential to meet daily energy demands and maintain homeostasis. A loss in both basal respiration and spare respiratory capacity may contribute to corneal epithelial damage and an increased risk of infectious keratitis in patients with diabetes. Given the importance of this vital tissue for vision, further studies are needed to identify the mechanisms that account for these mitochondrial changes.

## Figures and Tables

**Table 1:** Cellular oxygen consumption over time.

Duration of Culture (Days)	ATP-Linked Respiration (pmol/min/10,000 Cells)	Non-Mitochondrial O_2_ Consumption (pmol/min/10,000 Cells)	Proton Leak (pmol/min/10,000 Cells)
**Day 1**			
KGM	27.2 ± 1.7	18.2 ± 3.7	8.2 ± 0.6
MAN	35.3 ± 2.8 *	27.1 ± 2.2 **	7.8 ± 1.1
GLU	34.4 ± 2.2 *	24.2 ± 2.0	9.2 ± 0.9
**Day 5**			
KGM	22.7 ± 2.2	15.1 ± 1.5	6.9 ± 1.2
MAN	31.2 ± 3.4 *	17.4 ± 2.6	6.2 ± 0.6
GLU	17.0 ± 1.7 *^,†^	22.0 ± 1.0 *^,†^	4.2 ± 0.4 **^,††^
**Day 7**			
KGM	31.6 ± 4.8	15.6 ± 1.4	7.1 ± 1.1
MAN	39.3 ± 3.4 ***	21.5 ± 1.5 **	6.6 ± 0.5
GLU	43.3 ± 2.9 *	21.0 ± 0.6	7.2 ± 0.4
**Day 9**			
KGM	21.6 ± 1.9	11.9 ± 0.9	6.0 ± 1.4
MAN	24.6 ± 1.1 ****	16.6 ± 2.1 **	6.4 ± 0.5
GLU	37.4 ± 1.9 *^,†^	16.9 ± 1.1 **	6.8 ± 0.5
**Day 14**			
KGM	27.1 ± 5.0	17.2 ± 2.9	5.2 ± 1.4
MAN	16.5 ± 1.2 *	19.4 ± 1.2	3.5 ± 0.3 ******
GLU	9.3 ± 1.6 *^,†^	7.8 ± 1.3 *^,†^	4.0 ± 0.6 *****

* *p* < 0.001 compared to KGM, ** *p* < 0.05 compared to KGM, *** *p* = 0.004 compared to KGM, **** *p* = 0.008 compared to KGM, ***** *p* = 0.017 compared to KGM, ****** *p* = 0.039 compared to KGM, ^†^ *p* < 0.001 compared to MAN, ^††^ *p* < 0.05 compared to MAN.

**Table 2:** A summary of the metabolic changes from hyperglycemia compared to the KGM.

Duration of High Glucose Culture (Days)	Spare Respiratory Capacity	Extracellular Acidification Rate	Oxygen Consumption Rate	Metabolic Phenotype
1	Equal	Equal	Increase	Respiratory
5	Decrease	Decrease	Increase	Respiratory
7	Decrease	Decrease	Increase	Respiratory
9	Decrease	Decrease	Increase	Respiratory
14	Decrease	Equal	Decrease	Glycolytic

## Data Availability

The authors confirm that the data supporting the findings of this study are available within the article.
